# Soil Lead Concentrations in Dareta Village, Zamfara, Nigeria

**DOI:** 10.5696/2156-9614-9.23.190910

**Published:** 2019-08-22

**Authors:** Udiba U. Udiba, Ekom R. Akpan, Ekpo E. Antai

**Affiliations:** 1 Department of Zoology and Environmental Biology, University of Calabar, Calabar, Nigeria; 2 Institute of Oceanography, University of Calabar, Calabar, Nigeria

**Keywords:** environmental audit, lead, soil pollution, Dareta village, ecological risk, bioavailability/mobility

## Abstract

**Background.:**

Acute widespread lead poisoning took place in Zamfara State, Nigeria in 2010. Over 735 children were reported dead and thousands sickened by the neurotoxin. The source was traced to artisanal gold mining and processing in the villages. An immediate medical response protocol was developed to provide oral chelation therapy to the vulnerable population. In order not to compromise the efficacy of the chelation therapy, immediate remediation of the affected villages was carried out. An over 95% reduction in soil lead levels was reported immediately after the remediation exercise.

**Objectives.:**

The aim of the present study was to perform a general audit of soil lead concentrations, conducted between June and July 2013 in Dareta village (one of the most affected villages), to evaluate the soil pollution status of the village three years after the remediation exercise.

**Methods.:**

Soil samples were collected from residential compounds, cultivated farmlands and other common areas. Lead contents of the samples were determined using atomic absorption spectrophotometer (Shimadzu, model AA-6800, Japan) after wet digestion. Speciation of lead in soil was done following standard analytical methods.

**Results.:**

Mean soil lead concentrations for residential areas were 1029.42±98.50 mg/kg, 1523.99±201.00 mg/kg, 1404.57±141.00 mg/kg and 6724.68±84.00 mg/kg for residential compounds, market square, old grinding mills and new grinding mills, respectively. The concentrations exceeded both the Dutch target and intervention values and US Environmental Protection Agency limit for soil lead levels in residential areas. Based on the index of geo-accumulation, the ranking of intensity of lead (Pb) pollution of Dareta soils ranged from strongly polluted to extremely polluted, indicating they pose a range of moderate to very high potential ecological risk to the other components of the environment. At the current rate of accumulation, by the year 2025, soil Pb levels of Dareta common areas are expected to exceed the pre-remediation levels associated with several deaths.

**Conclusions.:**

Findings of this study indicate that the problem of lead poisoning is ongoing in Dareta village. Zamfara State authorities should address the challenge through sustained periodic assessment and cleanup of affected areas. Implementation of safer mining practices should be enforced immediately.

**Competing Interests.:**

The authors declare no competing financial interests

## Introduction

Population growth and economic development can have negative effects on the environment and surrounding ecosystem, creating significant environmental problems with far reaching consequences to land, water and air.^1^,^2^ Unregulated inputs of metal-contaminated materials into the natural environment pose a range of both short- and long-term environmental risks.^3^ With the exception of soils derived from weathering of parent rocks containing elevated levels of trace elements, the presence of elevated metals concentration in the environment is related to anthropogenic activities.^4^ Contaminants are continually discharged into the environment through industrial, agricultural, mining, and domestic processes.^1^,^5^ Mining, smelting and associated activities are some of the most important sources by which soil, plants and surface water are contaminated by metals. Toxic heavy metal contamination and subsequent pollution of the environment are issues of great concern on a global scale due to their numerous sources, widespread distribution and multiple effects on the ecosystem.^5^,^6^

Naturally occurring minerals, from which metals and gems can be profitably extracted, abound on earth. Mining involves extracting minerals and its constituents, especially gems and metals from surrounding earth and ores. Mining industries vary in size; some are large operations with modern ore processing facilities where crushing, washing and various chemical or physical separation processes are automated. Small-scale mining operations referred to as artisanal mining consists essentially of basic methods of extraction and processing relying mainly on manual labor and simple implements and methods. Artisanal mining is a means of livelihood to many; most of whom have little or no other possibility to make a living. Although low in profit, its contribution towards gold production and rural employment worldwide is highly significant. The Blacksmith Institute (now Pure Earth) estimated that about 10 to 20 million people across the globe work in artisanal gold mining activities.^7^ Even though small-scale gold mining is economically significant, it has been the target of serious opposition in recent years. This is due to the adverse environmental and social consequences with which it is associated. Some of these adverse effects include acid drainage, lead and mercury pollution.^8^ Toxic materials are often used by artisanal miners to separate gems and metals from the surrounding ores and silts. Mercury amalgamation, for instance, is the most common separating process in artisanal gold mining. In gold mining operations, artisans are commonly exposed to dangerous levels of harmful substances due primarily to lack of awareness or absence of safety and health regulation, or lack of enforcement of such regulations where available. Although most mined materials pose relatively small health risks, many of the heavy metals and naturally radioactive materials mined from the earth can be very dangerous to human health. In addition to the environmental and health risk that results from exposure to toxic materials, several artisanal mines are poorly constructed and are physically unstable. Such mines face potential shaft collapse, exposing miners to high risk of injury or fatality. These severe adverse consequences notwithstanding, artisanal gold mining is widespread and its practice continues to increase as the price for gold rises. A previous report found that between 2009 and 2011, the price of an ounce of gold was approximately doubled.^9^

**Abbreviations**
*ANOVA*: Analysis of variance
*Cf*: Contamination factor
*EF*: Enrichment factor
*Er*: Ecological risk factor
*IACF*: Individual average contamination factors
*I*_*geo*_: Index of geo-accumulation
*MF*: Mobility factor
*USEPA*: United States Environmental Protection Agency

Mining has been described as an extremely destructive practice that has serious negative impacts on the surrounding environment.^7^ Mining generates large amounts of mineral waste such as waste rocks or tailings. This is due to the fact that the materials mined are often surrounded by other ores and rocks. The material surrounding the ore that must be removed in order to access the desired mineral, metal or precious stone is referred to as waste rock. Tailings are waste materials that emanate from the “ore processing phase”. Tailings always contain toxic substances left over from the ore separating process together with small amounts of heavy metals (most of which are highly toxic).^7^ Additionally, in most cases, tailings contain materials and minerals that can lead to dangerous runoff, and when stored improperly, land and water contamination. In small-scale mines waste rock and tailing dump sites are usually not structurally sound. This allows contaminants to “spill out over the surrounding environment.”^10^

Lead is one of the toxic heavy metals that occur naturally in the earth's crust. Lead is mined and used for several materials such as pipes, paints, batteries and ceramics glaze. Lead is also commonly found in ores containing other frequently mined materials such as copper (Cu), gold, silver, zinc (Zn), and iron (Fe). There are many different mining operations worldwide that could potentially be responsible for releasing lead into the environment. Approximately 36 mining sites have been identified worldwide where lead contamination from mining processes poses a serious health risk to well over 12 million people, with the most impacted populations in South America and Africa.^7^

Acute widespread lead poisoning took place in Zamfara State, Nigeria in 2010. The rising price of gold in the international market sent villagers prospecting. Unfortunately, what the local miners found was gold ore laced with high concentrations of lead (concentrations as high as 10% in most cases). Consequently, thousands of villagers were exposed to mass lead contamination. Over 735 children died and thousands were sickened by the neurotoxin in what is believed to be the worst lead poisoning epidemic in modern history.^11^,^12^ In many areas of the village, including family homes and compounds, the soil lead concentration exceeded 100,000 mg/kg, far above the 400 ppm threshold considered acceptable for residential areas.^9^ An immediate medical response protocol was developed to provide oral chelation therapy to children between the ages of zero to five years old, pregnant women and breastfeeding mothers. In order not to compromise the efficacy of the chelation therapy, immediate remediation of the affected villages was carried out. The remediation project involved the removal of about five centimeters of contaminated topsoil from areas with soil lead levels above 1000 mg/kg. The excavated contaminated soil was then replaced with clean soil after previous investigations confirmed that the contamination was superficial. This was followed by burial of the removed contaminated surface soil in landfills. Areas with soil lead levels between 400–1000 mg/kg were simply covered with about 8 cm of clean soil and compacted.^9^,^13^ Immediately after the remediation exercise, soil lead levels in the village ranged from 81.65 to 684.27 mg/kg, indicating an over 95% reduction in soil lead levels.^13^ The present study focused on the audit of soil lead levels in Dareta village three years after the remediation exercise was carried out between June and July 2013.

## Methods

Zamfara State is located in the northwest geopolitical zone of Nigeria. Zamfara occupies a land mass of about 39 762 sq km and the Gusau is its capital. The state shares boundaries with Sokoto, Niger, Kebbi, Katsina and Kaduna State within the country and an international boundary with the Niger Republic.^14^

The climate of the northwestern Nigerian State is warm and tropical, with temperatures up to 38°C at the peak of the dry season (between March and April). The rains begin between mid-March and May, lasting for about six months until the end of October, while the extremely dry, cold and dusty wind that blows from the Sahara towards the western coast of Africa (Harmattan) lasts from December to February. The mean annual rainfall in the area fluctuates between 36 and 80 mm, varying slightly from the northern to the southern parts of the state. The vegetation of the state is made up of Sudan and Guinea Savannah. The Sudan Savannah is found in the northern, western and eastern part of the state. The Northern Guinea Savannah is found in the southern part of the state. This vegetation type is largely found in the Gusau, Tsafe and Anka Local Government Areas.^15^

Dareta village (the study area) is in Anka Local Government area and is located at 12°06′30′ N 5°56′00′ E with a total area of about 2746 sq km and population of about 142 280.^16^ Based on the 2006 Anka Local Government Area census data, the estimated population of Dareta village is 1033 and the number of children less than 5 years old (based on an estimated 20% of the population) is 207. The village is primarily populated by the Hausa and Fulani peoples. Until recently, following the discovery of gold mines, the main activity of the people of Dareta village was farming. Lately, artisanal mining activities has engaged a large percentage of the population.

### Collection of soil samples

Procedures for sample collection, preservation and preparation were adopted from Begum *et al*.^17^ Six residential compounds were randomly picked for the study. Soil samples were collected at the surface level (0–10 cm depth) in duplicates from the selected residential compounds using a soil auger. Six samples were obtained from different points in the market square which also act as a playground, six samples were collected from different ores were processed before the 2010 remediation exercise, and six samples from different points at the new grinding mills. A set of six samples collected from Basawa village, Zaria (a non-mining community) was used as control for residential areas. Five farms were also selected for the study. Farmland was divided into three sections. The three sections were designated as sampling points, 1, 2 and 3. Soil samples were collected from each of the three sampling points. Soil samples from each farm were thoroughly mixed together to obtain representative composite samples.

Soil conductivity was determined on site electronically using HACH conductivity / TDS meter (model 44600.00, USA). Soil pH was also determined on site electronically using a Zeal–tech digital pH meter (model 03112, India). Soil samples for lead determination were air dried, crushed and sieved with 2 mm mesh before wet digestion. One (1) g of a well-mixed sample from each sampling point was digested in a 250-ml glass beaker with 10 ml of concentrated nitric acid (HNO_3_), perchloric acid and hydrofluoric acid in the ratio 3:1:1 on a hot plate. After evaporating to near dryness, 10 ml of 2% HNO_3_ was added, filtered into 50 ml volumetric flask and then made up to the mark with distilled deionized water.^13^

Soil samples from the five selected farms were used for chemical speciation and were designated soil samples 1, 2, 3, 4 and 5. Sequential extraction was used to fractionate the heavy metal from the soil samples into six operationally defined groups or fractions: a water soluble fraction, exchangeable fraction, carbonate bound fraction, organic matter bound fraction, Fe-Mn oxide bound fraction and residual fraction, corresponding to Fraction 1, 2, 3, 4, 5 and 6, respectively.

The procedure was adopted from Kashem, which is a modification of a procedure by Tessier.^18^,^19^ Two (2) grams of soil sieved with 2-mm mesh was weighed into a 50-ml polycarbonate centrifuge tube and the following extractions were performed sequentially:

Fraction 1: Sample was extracted with 20 ml of distilled, deionized water for 2 hours at 20°C on a rolling table.
Fraction 2: The residue from Fraction 1 was extracted with 20 ml of 1 M ammonium acetate (NH_4_OAc) (pH 7) for 2 hours at 20°C on a rolling table.
Fraction 3: The residue from Fraction 2 was extracted with 20 ml of 1 M NH_4_OAc (pH 5) for 2 hours at 20°C on a rolling table.
Fraction 4: The residue from Fraction 3 was extracted with 20 ml of 0.04 M hydroxylamine hydrochloride (NH_2_OH.HCl) in 25% acetic acid (vol/vol) at pH 3. The reaction time was 6 hours in a water bath at 80°C with occasional shaking.
Fraction 5: The residue from Fraction 4 was extracted with 15 ml of 30% hydrogen peroxide adjusted to a pH of 2, reaction time 5.5 hours in a water bath at 80°C with occasional shaking. After cooling, 5 ml of 32 M NH_4_OAc in 20% (vol/vol) HNO_3_ was added, sample was shaken on a rolling table for 0.5 hours at 20°C and finally diluted to 20 ml with distilled deionized water.
Fraction 6: The residue from Fraction 5 was extracted with 7 M HNO_3_ in a water bath at 80°C with occasional shaking. The reaction time was 6 hours. Duplicates analysis was performed for each sample.

### Sample analysis

Lead concentrations in the digests and extracts from sequential extraction were determined by atomic absorption spectrophotometry (Shimadzu, model AA-6800, Japan) equipped with Zeeman background correction and graphite furnace at the National Research Institute for Chemical Technology, Zaria, Nigeria. The calibration curve was prepared by running different concentrations of the standard solution (lead II nitrate in nitric acid). The instrument was then set to zero by running the respective reagent blanks and lead (Pb) concentration was determined at a wavelength of 283.3 nm. Average values of three replicates were taken for each determination. Data obtained were subjected to statistical analysis.

### Analytical quality assurance

Appropriate quality assurance procedures and precautions were taken to ensure the authenticity of the results. Samples were carefully handled to avoid cross-contamination. Glassware was properly cleaned and deionized water was used throughout the study. Reagents used in the present study include HNO_3_ (Riedelde Haen, Germany), hydrochloric acid, NH_4_OAc, hydroxylammonium chloride and hydrogen peroxide (Sigma-Aldrich, Germany) and hydrogen fluoride and perchloric acid (British Drug House Chemicals Limited, England) were all of analytical grade.

In order to check the reliability (accuracy and precision) of the analytical method employed for Pb determination, one blank and combined standards were run with every batch of samples to detect background contamination and monitor consistency between batches. The result of the analysis was validated by digesting and analyzing Standard Reference Material (Lichens coded IAEA-336) following the same procedure. 13 The analyzed values and the certified reference values of the elements determined were very close, indicating good reliability *(Table 1).*

**Table 1:** Results of Analyzed Reference Material (Lichen IAEA - 336) Compared to the Certified Reference Values (mg/kg)

**Element (mg/kg)**	**Pb**	**Cd**	**Cu**	**Mn**	**Zn**
**Analyzed value**	5.25	0.140	4.00	55.78	29.18
**Reference value**	4.2–5.5	0.1–2.34	3.1–4.1	56–70	37–33.80

Abbreviation: Cd, cadmium.

The pre-industrial reference level and the toxic response factor determined by Hakanson and used for the computation of the contamination factor and the ecological risk factor in this study are given in Table 2.^26^,^20^

**Table 2:** Pre-Industrial Reference Level (μg/g) and Toxic-Response Factor Used for the Computation of Contamination Factor and Ecological Risk Factor

**Elements**	**Hg**	**Cd**	**As**	**Cu**	**Pb**	**Cr**	**Zn**
**Pre-industrial reference level**	0.25	1.0	15	50	70	90	175
**Toxic-response factor**	40	30	10	5	5	2	1

Abbreviations: Hg, mercury; Cd, cadmium; As, arsenic; Cr, chromium.

### Statistical analysis

The test for normality was carried out using the Shapiro-Wilk test and the Z-score test was used to check for outliers.

### Analysis of variance test

Having passed the test for normality and outliers, data collected were subjected to a statistical test of significance using the analysis of variance (ANOVA) test to assess significant variation in soil Pb levels across the sampling locations. Probabilities of less than 5% (p < 0.05) were considered statistically significant.

### Independent t-test

Independent t-test was used to compare soil lead levels immediately after the remediation exercise and the present study. Soil Pb levels in the study area and control (Zaria) were also compared using the independent t-test. Probabilities of less than 5% (p < 0.05) were considered statistically significant. All statistical analyses were performed using SPSS software version 17.00 for Windows.

### Bioavailable and non-bioavailable fractions

The bioavailable fraction was computed from the speciation according to Ogunfowokan *et al.* as the sum of the water soluble fraction, exchangeable fraction and carbonates-bound fraction as given in Equation 1.^21^ The non-bioavailable fraction was calculated as the sum of the Fe-Mn oxide bound fraction, organic matter bound fraction, and the residual fraction as given in Equation 2.

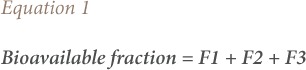


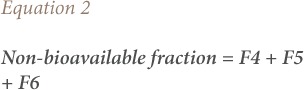
where, F1 is the water-soluble fraction, F2 is the exchangeable fraction, F3 is the carbonates-bound fraction, F4 is the Fe-Mn oxide bound fraction, F5 is the organic matter bound fraction, and F6 is the residual fraction.

### Mobility factor

Mobility factor (MF) is described as the index of potential mobility of metal ions in soil. In this study, MF was determined on the basis of the absolute and relative values of fractions (water soluble fraction, exchangeable fraction and carbonates-bound fraction) that are weakly bound to soil components using the formula expressed in Equation 3.^24^,^22^

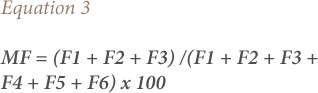
where, F1 is the water-soluble fraction, F2 is the exchangeable fraction, F3 is the carbonates bound fraction, F4 is the Fe-Mn oxide-bound fraction, F5 is the organic matter-bound fraction, and F6 is the residual fraction.

Some Pb forms (extracted in the carbonates-bound fraction) are observed to be more strongly bound to soil components (less mobile) than those extracted in the water-soluble fraction and exchangeable fraction, and the above-mentioned index therefore describes potential mobility. Furthermore, when the pH and redox conditions are favorable, the Fe-Mn oxide-bound Pb may be soluble and remobilized into other components of the environment (water, plants and biota). Low MF values (below 40%) are interpreted as indicators of stability (non-bioavailability), while high MF values are interpreted as indicators of biological availability.^21^

### Individual average contamination factor

Individual average contamination factors (IACF) were calculated from the result of the sequential extraction by dividing the sum of the first five fractions (water soluble fraction, exchangeable fraction, carbonate bound fraction, Fe-Mn oxide bound fraction and organic matter bound fraction) by the residual fraction for soil *(Equation 4)* and reflects the risk of contamination of soil by Pb.^22^ The higher the levels of the mobilizable fraction (i.e. the water soluble, exchangeable, carbonate bound, Fe-Mn oxide bound and bound to organic matter) in the soil, the higher the potential risk of soil contamination.^23^,^22^

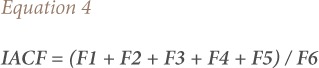
where, F1 is the water-soluble fraction, F2 is the exchangeable fraction, F3 is the carbonates-bound fraction, F4 is the Fe-Mn oxide-bound fraction, F5 is the organic matter-bound fraction, and F6 is the residual fraction.

### Average factor of accumulation

The average factor of accumulation of Pb in Dareta soil from 2010 when the remediation exercise was carried out to July 2013 was computed according to Uzairu *et al.* as the ratio of average concentration at a given location in the present study to the average concentration at the same location in 2010.^22^

### Contamination factor

Contamination factor (Cf) was used to describe soil contamination as suggested by Hakanson, and Qingjie *et al.*^24^,^19^ The formula below was used to calculate the contamination factor:

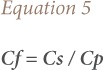
where, Cs is the mean content of lead from at least 5 sample sites and C_p_ is the pre-industrial reference level for lead *(Table 2)*.

### Ecological risk factor

An ecological risk factor (Er) used to quantitatively express the potential ecological risk posed by a given contaminant suggested by Hakanson and Qingjie *et al.* was also used to express the potential ecological risk posed by the heavy metal (Pb) in the Dareta ecological geochemical environment.^24^,^20^ The formula below was used to calculate the Er.

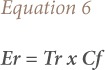
where, is the toxic-response factor for a given substance. The toxic-response factors of heavy metals including arsenic are presented in *(Table 2)*.^20^

### Enrichment factor

The enrichment factor (EF) of an element was initially developed to speculate the origin of the element in the atmosphere, precipitation and sea water, but it was later extended progressively to the study of soil, peat tailings, lake sediments and other environmental materials.^24^ The following formula was used to calculate the enrichment factor:

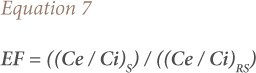
where, C_e_ is the concentration of an element in the sample of interest or the selected reference sample, and C_i_ is the concentration of an immobile element in the sample or the selected reference sample. So, (C_e_ / C_i_)_S_ is the heavy metal to immobile element ratio in the samples of interest, and (C_e_ / C_i_)_RS_ is the heavy metal to immobile element ratio in the selected reference sample.^24^

The selected reference sample is usually an average crust or a local background sample. Samples from Zaria, used as the control for this study, were selected as the reference sample.^25^ The immobile element is usually taken to be aluminum, lithium, scandium, zirconium, or manganese, and manganese was used in the present study.^24^

### Index of geo-accumulation

An index of geo-accumulation (I_geo_) was used to determine and define Pb contamination in soil by comparing current concentrations with pre-industrial levels. It was calculated following the equation below.

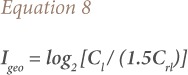
where, C_l_ is the measured concentration of the examined metal (Pb) in soil, and C_rl_ is the geo-chemical background concentration or reference value of the metal (Pb). The factor 1.5 was introduced because of possible variation in the background value for a given metal in the environment as well as very small anthropogenic influences on the value.^24^

### Bioavailability factor

The bioavailability factor of heavy metals in plants, also known as the bioavailability index, was calculated according to Malik *et al.* using Equation 9.^26^

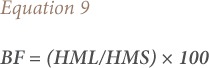
where, BF is the bioavailability factor, HM_L_ is mg of heavy metal per kg of plant leaves and HM_S_ is the total content of heavy metal per kg of soil. In the present study, the reference values used for the estimation of single pollution indices are divided into background levels and threshold pollution values. The background levels (*Table 2*), suggested by Hakanson, were used in the estimation of Cf, I_geo_, and Er.^20^ Values from the analysis of samples from Zaria (a non-mining area, used as control for this work) were used as the threshold pollution values for the estimation of enrichment factor. A single pollution index value > 1.0 indicates that it is polluted when threshold value was referred.^24^

### Biological concentration factor, translocation factor and biological accumulation coefficient

The biological concentration factor was calculated as the metal concentration ratio of plant roots to soil as given in Equation 10. The translocation factor was described as the ratio of heavy metals in the plant shoot to that in the plant root *(Equation 11)*. The biological accumulation coefficient was calculated as the ratio of heavy metals in the plant shoot to that in soil *(Equation 12)*.^26^

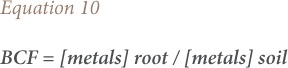


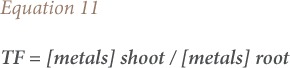


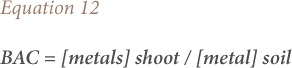
where, BCF is the biological concentration factor, TF is the translocation factor and BAC is the biological accumulation coefficient.

## Results

The results obtained from the determination of soil Pb levels across the different sampling points in Dareta village are presented in Table 3. The comparison of 2010 and 2013 mean soil Pb levels cross the various sampling locations is shown in Figure 2 and the results of ecological risk assessment of lead in Dareta soil using single pollution indices are presented in Table 4.

**Figure 1:**
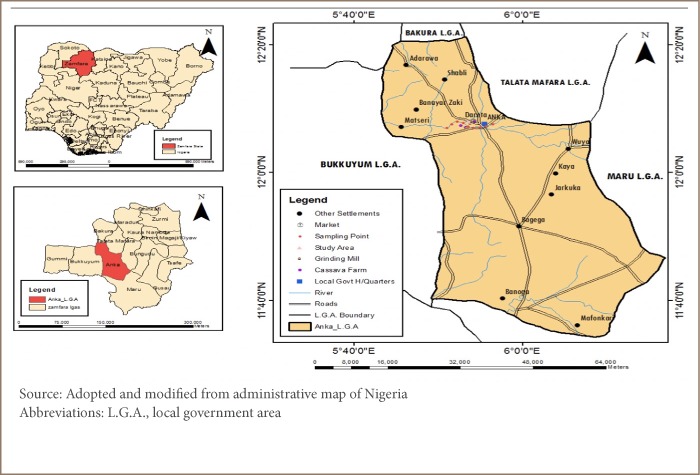
Map of Anka Local Government Area of Zamfara State, Nigeria showing Dareta Village with sampling points

**Figure 2:**
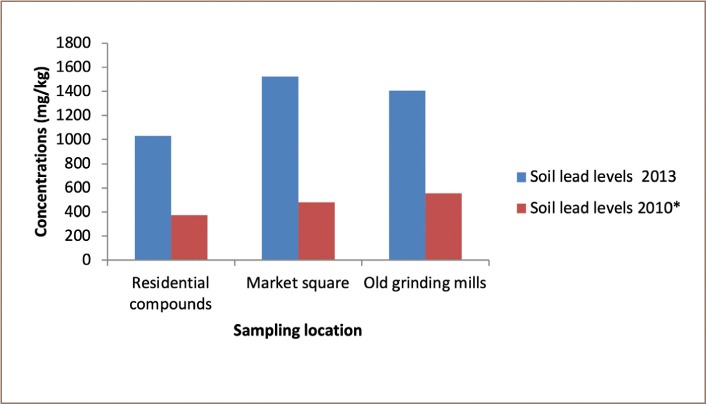
Comparison of mean soil Pb levels across Dareta village in 2010 and 2013

**Table 3:** Determination of Soil Pb Levels (mg/kg) Across Sampling Points in Dareta Village, Anka, Nigeria

**Sample Numbers**	**Residential compounds**	**Market square**	**Old grinding mills**	**New grinding mills**	**Control**
**1**	3185.47	1787.86	3877.01	9010.34	58.58
**2**	585.81	1145.48	497.42	7677.26	86.41
**3**	389.12	1520.67	449.54	6941.09	48.94
**4**	346.77	1549.08	952.20	4544.71	108.78
**5**	955.89	1458.14	2302.33	4471.86	49.48
**6**	713.44	1682.69	348.90	7702.84	60.56
**Mean ± SD**	1029.42±98.50^a^	1523.99±201.00^a^	1404.57±41.00^a^	6724.68±84.00^b^	68.79±23.86^c^
**Range**	346.77–3185.47	1145.48–1787.86	348.90–3877.01	4471.86–9010.34	48.94–108.78
**US EPA Limits^d^**	400 mg/kg	400 mg/kg	400 mg/kg	400 mg/kg	400 mg/kg

Mean with the same superscripts indicates significant (p < 0.05, ANOVA) difference.

^d^ Adopted from Lead Pollution and Poisoning Crisis Environmental Emergency Response Mission: Zamfara State, Nigeria September/October 2010^14^

Abbreviations: SD, standard deviation; US EPA, US Environmental Protection Agency.

**Table 4:** Ecological Risk Assessment of Lead in Dareta Soil Using Single Pollution Indices

**Location/pollution index**	**Residential compounds**	**Market square**	**Old grinding mills**	**New grinding mills**	**Mean**
**Contamination factor**	14.71	21.77	20.07	96.06	38.15
**Ecological risk factor**	72.55	108.85	100.35	480.45	90.8
**Enrichment factor**	5.37	6.50	6.43	28.35	11.66
**Index of geo-accumulation**	3.29	3.86	3.74	6.00	4.22
**Average accumulation factor**	2.78	3.18	2.54	-	2.83

Table 3 indicates that the order of detection of lead in residential compounds and Dareta common areas was, new grinding mills > market square > old grinding mills > residential compounds. Lead concentrations ranged between 346.77 mg/kg – 9010.34 mg/kg. The lowest concentration (346.77 mg/kg) was recorded in the residential compound and the highest concentration (9010.34 mg/kg) in the new grinding mills.

The overall mean level in the village was found to be 2670±155.42 mg/kg, while the average level across sampling locations in the village was 1029.42±98.50 mg/kg, 1523.99±201.00 mg/kg, 1404.57±41.00 mg/kg, 6724.68±84.00 mg/kg and 68.79±23.86 mg/kg for residential compounds, market square, old grinding mills, new grinding mills and the control, respectively.

A positive correlation was observed between Pb levels in the residential compounds and Pb levels in the market square (r = 0.568), old grinding mills (r = 0.908), and new grinding mills (r = 0.575). However, only the correlation between residential compounds and the old grinding mills was found to be statistically significant at a 95% confidence level. Lead levels in soils in the market square correlated positively with those at the old grinding mills (r = 0.489) and new grinding mills (r = 0.242). The lead concentration in the old grinding mills also correlated positively with the new grinding mills (r = 0.193), but the correlations were not statistically significant (p > 0.05). A negative correlation was observed between the control and residential compounds (r = −0.314), market square (r = −0.317), old grinding mills (r = −0.291) and new grinding mills (r = −0.285). The statistical analysis showed that the difference in soil lead levels across the sampling locations was statistically significant (ANOVA, p < 0.05), with Pb levels in the new grinding mills significantly (p < 0.05) higher than Pb levels in the residential compounds, market square and old grinding mills. Soil Pb levels in residential compounds and the old grinding mills in the present study were found to be relatively higher than their corresponding 2010 levels, but the differences were not statistically significant (p > 0.05). A statistically significant (p < 0.05) difference in soil lead levels at the market square was observed between the present study and the remediation level in 2010, with soil lead levels in the present study significantly higher than the 2010 levels. The difference in soil lead concentration in the study area was found to be significantly higher than in the control.

Table 4 shows the single pollution indices used to assess the quality of the geochemical environment of Dareta village. Soils from the new grinding mills recorded the highest contamination factor (96.06) and residential compounds the lowest contamination factor (14.71). The trend for contamination factor was new grinding mills > market square > old grinding mills > residential compounds. The Er for the new grinding mills was 480.45, while that of the residential compounds was 72.55. The ecological risk factor also followed the same trend of new grinding mills > market square > old grinding mills > residential compounds. The EF was 5.37, 6.50, 6.43, and 28.35 for residential, market square, old grinding mills and new grinding mills, respectively. The I_geo_ was 3.29, 3.86, 3.74 and 6.00 for residential, market square, old grinding mills and new grinding mills, respectively. The average accumulation factor was 2.78 for residential compounds, 3.18 for market square and 2.54 for old grinding mills.

### Chemical speciation of lead

**Figure 3:**
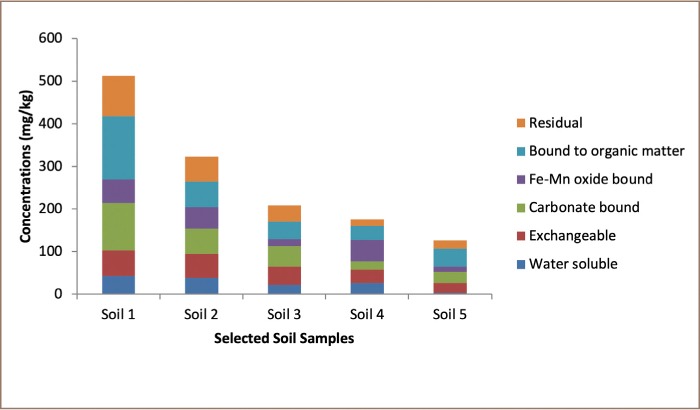
Spatial distribution of extractable lead across forage and farm soils in Dareta

Physicochemical parameters (soil pH and conductivity) of Dareta soil is presented in Table 5. Chemical speciation/percentage distribution of extractable lead in Dareta soil, and potential MF and IACF are presented in Table 6. The spatial distribution of extractable lead across Dareta soil is shown in Figure 3. The concentration and percentage concentration of bioavailable and non-bioavailable fractions of lead in Dareta soil are presented in Table 7.

**Table 5:** Result Obtained from the Determination of Soil pH and Conductivity in Dareta Village

**Physicochemical parameters**	**Soil 1**	**Soil 2**	**Soil 3**	**Soil 4**	**Soil 5**	**Mean**
**pH**	5.85	6.29	6.59	6.64	6.8	6.43
**Conductivity (μS/cm)**	1200	700	400	290	300	578

**Table 6:** Chemical Speciation, Potential Mobility Factors and Individual Average Contamination Factors of Dareta Soil

**Soil sample/fraction**	**Soil 1**	**Soil 2**	**Soil 3**	**Soil 4**	**Soil 5**	**Mean**
**Water soluble**	41.94	37.96	21.94	25.58	2.97	26.08±15.36 (10%)
**Exchangeable**	61.37	56.15	42.97	32.41	23.31	43.24±15.89 (16%)
**Carbonate bound**	110.82	59.11	46.96	18.76	25.58	52.25±36.53 (19%)
**Bound to Fe-Mn oxide**	55.24	50.47	17.39	51.16	13.08	37.47±20.43 (14%)
**Bound to organic matter**	147.88	60.92	40.13	31.84	42.07	64.57±47.77 (24%)
**Residual**	94.43	58.76	38.99	15.34	18.76	45.26±32.55 (17%)
**Pseudo total**	511.68	323.37	208.38	175.09	125.77	268.86±13.06
**Total lead**	692.91	457.13	235.73	187.75	130.91	340.89±232.4
**Mobility factors**	41.85	47.34	53.29	43.83	41.23	45.51±4.96
**Individual average contamination factors**	4.42	4.5	4.34	10.41	5.7	5.87±2.60

Note: values are in mg/kg except MF and IACF which are ratios.

**Table 7:** Total Lead Content, Concentration and Percentage Concentration of Bioavailable and Non-Bioavailable Fractions (mg/kg) of Dareta Soil

**Sampling Station**	**Concentration bioavailable**	**Concentration non-bioavailable**	**Total lead content**	**% Bioavailable**	**% Non-bioavailable**
**Soil 1**	214.13	297.55	511.68	41.85	58.15
**Soil 2**	153.22	170.15	323.37	47.38	52.62
**Soil 3**	111.87	96.51	208.38	53.69	46.31
**Soil 4**	76.75	98.34	175.09	43.83	56.17
**Soil 5**	51.86	73.91	125.77	41.23	58.77
**Mean**	121.57	147.29	268.86	45.60	54.40

The physicochemical parameters of Dareta soil as presented in Table 5 shows that pH ranged from 5.85 to 6.80 with highest value recorded in soil 5 and lowest value recorded in the soil 1. The mean pH value was 6.43 ± 0.38. Electrical conductivity of the soils ranged between 290 μS/cm - 1200 μS/cm with a mean value of 578±0.39 μS/cm. The highest value was recorded in soil sample 1, while the lowest value was recorded in soil sample 4.

Table 6 and Figure 3 revealed that Pb in Dareta soil was largely associated with organic matter and carbonate fractions with a significant amount in the residual and exchangeable fraction. The overall mean levels of Pb (mg/kg) in the fractions were: water soluble fraction (26.08±15.36 mg/kg (10%); exchangeable fraction (43.24±15.89 mg/kg (16%); carbonate bound fraction (52.25±36.53 mg/kg (19%); Fe-Mn bound fraction (37.47±20.43 mg/kg (14%); organic matter bound fraction (64.57±47.77 mg/kg (24%); and residual fraction (45.26±32.55 mg/kg (17%). Lead levels occurred in the order of organic matter bound > carbonates bound > residual > exchangeable > Fe-Mn bound fraction > water soluble.

Extractable Pb followed the trend: soil 1 > soil 2 > soil 3 > soil 4 > soil 5 which correspond to the order of increasing distance from residential areas, indicating that Pb levels decreases with increasing distance from village residential areas. The values of potential MF, with an overall mean of 45.51±4.96, decreased in the following order: soil 3 > soil 2 > soil 4 > soil 1 > soil 5. Apart from reflecting the levels of water soluble, exchangeable and carbonates-bound lead in this study, high MF values were an indication of the lability and biological availability (bioavailability) of the toxic metal. The values of IACF, with an overall mean of 5.87±2.60, decreased in the following order: soil 4 > soil 5 > soil 2 > soil 1 > soil 3.

Table 7 indicates that lead was associated with water soluble, exchangeable and carbonates bound (bioavailable) fractions corresponding to 45.60% of total extractable lead (pseudo total), and Fe-Mn oxide bound, organic matter bound and residual (non-bioavailable) fractions corresponding to 54.40% of total extractable lead. The mean bioavailable Pb relative to total Pb contents for the five soils followed the sequence: soil 1 > soil 2 > soil 3 > soil 4 > soil 5. The mean non-bioavailable Pb followed the order: soil 1 > soil 2 > soil 4 > soil 3 > soil 5. A significant (p < 0.01) positive correlation was observed between total soil Pb concentration and extractable Pb concentration (bioavailable fraction, r = 0.977 and non-bioavailable fraction r = 0.988) and between total soil lead concentration and soil conductivity (r = 0.966).

On the other hand, total lead concentration was found to correlate (r = −0.945) negatively with soil pH, and the correlation was significant at 95% confidence levels. Soil pH was found to correlate significantly with extractable lead concentration (bioavailable fraction, p < 0.01, r = −0.984 and non-bioavailable fraction, p < 0.05, r = −0.904), indicating that an increase in pH is associated with a decrease in the extractable metal concentrations. A strong negative correlation (r = −869) was observed between soil conductivity and soil pH. The correlation was statistically significant at a 95% confidence level. Significant, positive correlations were observed between soil conductivity and the extractable fractions (bioavailable fraction, p < 0.05, r = 0.907 and non-bioavailable fraction, p < 0.05, r = 0.982), indicating that extractable lead concentration increases as conductivity increases. The concentration of bioavailable lead in soil was found to correlate significantly (p < 0.05, r = 0.954) with the concentration of non-bioavailable lead, suggesting that the same lead source may be responsible for their presence at the concentrations determined.

## Discussion

The results of the analysis of the physicochemical properties of soil quality indicated that the soils of the present study area were slightly acidic in nature with a mean pH of 6.43 and relatively high electrical conductivity (290 μS/cm -1200 μS/cm). Metal solubility, mobility and bioavailability in soils increases with increasing acidity. The slight acidity of Dareta soil may enhance the solubility and mobility of lead, and may also play a vital role in its bioavailability and transfer into the other components of the environment, including the food chain.^27^ Metal solubility, mobility and bioavailability in soils are predominantly controlled by pH, cation exchange capacity, electrical conductivity, organic carbon content and the oxidation state of the metal.^26^, ^28^ Similar pH values (6.04 and 5.6) were recorded for cultivated and uncultivated soils in Yuri, Turkey. These values were associated with high mobility of metals including lead.^27^ A pH range of 4.7 - 6.5 was reported for different locations in Japan, where mobility and bioavailability of lead was shown to increase with decreasing pH values.^18^ A higher mean pH value of 8.2 and electrical conductivity value of 7.7 dS/m was reported in the Segura River valley, Spain, and these values were associated with high stability of the toxic metal.^28^

### Total lead concentration

The mean soil Pb levels of Dareta village common areas *(Table 3)* were all found to be above 400 mg/kg, which is the United States Environmental Protection Agency (USEPA) limit for soil lead levels in residential areas.^9^ Nigeria has no standards for soil lead levels, but the Department of Petroleum Resources adopts the Dutch standards for the assessment of soil pollution in the country. The Dutch soil remediation policy uses target and intervention values to assess soil contamination. The remediation intervention value, which is used to indicate the Pb level at which the functional properties of the soil support human, animal and plant life are seriously threatened or impaired, is 530 mg/kg. This value represents soil lead concentration above which the soil is said to be seriously contaminated. The target value (85 mg/kg) indicates the soil lead level for sustainable soil quality and is the soil Pb level that must be attained to fully recover all the functional properties of the soil for human, animal and plant life to thrive. The target value is the benchmark for environmental quality on the assumption of negligible risk to the ecosystem.^30^ Soil lead levels recorded in residential compounds and Dareta common areas were also found to be above both the Dutch target and intervention values.

Two out of six of the residential compounds studied recorded lead levels within the USEPA limit for soil lead levels in residential areas. Residential compound 1 recorded lead levels almost 8 times the permissible limit, while residential compounds 5 and 6 recorded lead levels around twice the limit. Residents of compounds with high soil lead levels are exposed to serious health risks, particularly young children who spend most of their time in the contaminated compounds and are chronically poisoned through their normal hand to mouth behavior as they play in the dust.^9^,^13^,^15^ All the sampling points in the market square, which also acts as the village playground, recorded soil lead levels that exceeded both the Dutch target and intervention values. The recorded soil lead levels were about 3 to 4 times the US EPA limit *(Table 3)*. Similarly, all the sampling points at the old and new grinding mills had soil lead levels well above Dutch target and intervention values, and the USEPA limit of 400 mg/kg *(Table 3)*.

Generally, the higher soil Pb levels observed in the residential compounds and Dareta common areas in the present study when compared to the corresponding value in 2010 immediately after the remediation exercise suggest that not all of the sources of lead in the village were completely identified and addressed of during the remediation exercise or that mining and processing activities have been carried out after the remediation exercise. The government of Nigeria initiated an immediate ban on mining activities in the area at the peak of the lead pollution and poisoning crisis in the state without providing alternative means of earning a comparable wage. This action only promoted illegal mining activities which are even more dangerous, as ore processing is currently carried out within the confines of residential compounds to keep from view of the authorities.

The high spatial variation in soil lead levels observed across the study area suggests an anthropogenic influence, as this metal may not have entirely originated from natural processes or crustal materials. The significantly higher soil Pb concentration in soil samples from the mining area (Dareta village) compared to the non-mining area (Zaria, control) may be a clear indication of the presence of toxic metal pollution due to mining activities. The positive correlation observed between soil lead levels in the residential compounds and market square, old grinding mills and new grinding mills indicates that increasing lead levels in the residential compounds are associated with increasing Pb concentrations, suggesting that the same source is responsible for the presence of Pb at the determined concentration in these locations. The negative correlation observed between the control and residential compounds, market square, old grinding mills and new grinding mills suggests that different sources are responsible for the presence of lead in Dareta village and Zaria (control). In previous studies, a range of 10.10 mg/kg – 73.83 mg/kg was reported for top soil (0–10 cm) in Umuahia, Nigeria and 35.9 – 306.7 μg/g during the dry season and 24.00 – 316.14 μg/g during the wet season in Yauri, Nigeria.^3^,^31^ In the Segura River valley in Spain, a lead concentration ranging from 8.9 – 34.5 mg/kg was reported.^29^ A study done in contaminated sites in Florida reported a total lead concentration in soil ranging from 90 mg/kg – 4100 mg/kg.^32^ A range of 81.65 – 684.27 mg/kg dry weight was reported in Dareta village immediately after the remediation exercise.^13^ The soil lead levels recorded in the present study were found to be higher than those reported in the literature, as well as the global baseline level of lead (20 mg/kg) in uncontaminated surface soils.^32^,^33^

### Ecological risk assessment

The average accumulation factor was found to be 2.78 for residential compounds, 3.18 for the market square and 2.54 for the old grinding mills, indicating that the mean soil lead level of residential compounds was 2.78 times its value in 2010, corresponding to an 178% increase, while those of market square and old grinding mills were 3.18 and 2.54 times their values in 2010, corresponding to 218% and 154% increases, respectively. This implies that with all of the conditions remaining constant, 3 years from the present study (July 2016), the average Pb levels of Dareta common areas may rise to approximately 2861.78 mg/kg, 4846.29 mg/kg and 3567.61 mg/kg for residential compounds, market square and old grinding mills, respectively. The pre-remediation soil Pb levels ranged between 60 000 mg/kg – 100 000 mg/kg.^14^ This level was associated with several deaths, particularly among children between 0 – 5 years of age who are uniquely susceptible to lead poisoning. From the average accumulation factors it is estimated that by the year 2025, soil lead levels of Dareta common areas will exceed the pre-remediation soil lead levels and will hit all-time high values of 6 1485.37 mg/kg, 15 5844.18 mg/kg and 5 4460.61 mg/kg for residential compounds, market square and old grinding mills, respectively *(Table 8)*.

**Table 8:** Estimated Soil Lead Concentration Based on Average Accumulation Factor

**Year**	**Residential compounds**	**Market square**	**Old grinding mills**	**Range**
**2010**	370.63	479.46	553.42	370.63–553.42
**2013**	1029.42	1523.99	1404.57	1029.42–1523.99
**2016**	2861.78	4846.29	3567.61	2861.78–4846.29
**2019**	7955.78	15411.20	9061.73	7955.78–15411.20
**2022**	22117.04	49007.60	28016.78	15411.20–49007.60
**2025**	61485.37	155844.18	58462.61	58462.61–155844.18

Metal Cf was also applied to evaluate the anthropogenic contribution of lead in Dareta soil. The contamination factor was 14.71 for the residential compound, 21.77 for the market square, 20.07 for the old grinding mills and 96.06 for the new grinding mills, corresponding to very high contamination. The following terminologies were used to explain Cf: “Cf < 1, low contamination factor; 1 ≤ Cf < 3, moderate contamination factor; 3 ≤ Cf < 6, considerable contamination factors; and Cf ≥ 6, very high contamination factor”.^24^ The Cf for this study is very high, suggesting anthropogenic contributions of lead in Dareta soil.

The ecological risk factor was found to be 72.55 for residential compounds, 108.85 for the market square, 100.35 for old grinding mills and 480.45 for new grinding mills. Ecological risk factor in this case represents the sensitivity of various biological communities to Pb contamination and illustrates the potential ecological risk caused by the heavy metal. The following terminologies were used to explain the ecological risk factor: Er< 40, low potential ecological risk; 40 ≤ Er < 80, moderate potential ecological risk; 80 ≤ Er < 160, considerable potential ecological risk; 160 ≤ Er < 320, high potential ecological risk; and Er ≥ 320 very high potential ecological risk.^24^ Lead contaminations of residential compounds in Dareta village therefore pose moderate potential ecological risk to the other components of the environment. Lead contamination of the market square and old grinding mills pose considerable potential ecological risk, while that of the new grinding mills poses very high potential ecological risk to the other components of the environment.

Measurement of EF is an essential part of geochemical studies. Enrichment factor is generally used to speculate the origin of metals in the soil environment. It differentiates between metals originating from anthropogenic (non-crustal) and geogenic (crustal) sources and assesses the degree of metal contamination. Enrichment factor > 1 implies soil contamination, 0 < EF < 10 is an indication for natural origin (initial soil or parent rock), while those > 10 are considered to be from anthropogenic sources.^34^ Table 4 indicates that the mean EF for lead in the study was 11.66. This implies that anthropogenic sources are responsible for lead pollution in Dareta. Lead EF was found to be 5.37 for residential compounds, 6.50 for the market square, 6.43 for old grinding mills and 28.35 for new grinding mills, corresponding to significant enrichment for residential, market square and old grinding mills, and very high enrichment for new grinding mills. Five contamination categories were used to describe EF: “EF < 2, depletion to mineral enrichment; 2 ≤ EF < 5, moderate enrichment; 5 ≤ EF < 20, significant enrichment; 20 ≤ EF < 40, very high enrichment; and EF > 40, extremely high enrichment”.^24^

Based on the values of I_geo_, the ranking of intensity of lead pollution of Dareta soil was as follows: new grinding mills > market square > old grinding mills > residential compounds. In Muller's model, seven classes of I_geo_ were determined: “I_geo_ ≤ 0, class 0, unpolluted; 0 < I_geo_ ≤ 1, class 1, from unpolluted to moderately polluted; 1 < I_geo_ ≤ 2, class 2, moderately polluted; 2 < I_geo_ ≤ 3, class 3, from moderately polluted to strongly polluted; 3 < I_geo_ ≤ 4, class 4, strongly polluted; 4 < I_geo_ ≤ 5, class 5, from strongly polluted to extremely polluted; and I_geo_ > 5, class 6, extremely polluted”.^24^ Following this model, residential compounds, market square and old grinding mills may be classified as strongly polluted, and new grinding mills extremely polluted. The average value of I_geo_ across Dareta soil was found to be 4.2, corresponding to pollution intensity ranging from strongly polluted to extremely polluted soil.

On the other hand, the results of the analysis of total lead content of Dareta soils reveals that farmlands in Dareta village were not contaminated, as soil Pb levels were within the USEPA limit for lead in soil (400 mg/kg) and the Dutch intervention value (530 mg/kg), the only exception being sample soil 1. This finding is consistent with the joint United Nations Environment Programme/Office for the Coordination of Humanitarian Affairs (UNEP/OCHA) report on the Zamfara lead pollution and poisoning crisis. The joint UNEP/OCHA environment unit reported in 2010 that lead pollution in Dareta is confined to areas where the processing of the lead rich gold ore has taken place and has not spread to other areas such as farmlands.^14^ Pollution indices based on total metal content (as discussed above) are good tools and have been used to express the degree of soil contamination in the residential compounds and village common areas of Dareta village.

### Chemical speciation

Although the total concentration of Pb in soil has been very useful for the characterization of the intensity of contamination of the toxic metal, the use of total soil Pb content as a criterion for assessing the potential effect of soil Pb contamination implies that all forms of the metal in soil have equal impact on the environment. This assumption does not have a sound defense.^19^ It is therefore necessary to determine not just how much of the contaminant is present, but also to ascertain the chemical form of the contaminant in addition to the total amount that is bioavailable for a full risk assessment. Toxicity, bioavailability, biological transportation and distribution, and thus, the ultimate impact of the toxic metal on the environment can be detected by the type of element in a sample.^21^ In this study, about 10% of extractable lead was present in the water soluble phase and 16% in the exchangeable phase. The water soluble and exchangeable fractions are not only considered as immediate nutrient reservoirs for plants, but generally as readily available for biogeochemical cycles in the ecosystems. Lead associated with carbonates (19%) is susceptible to pH changes and may be regarded as potentially bioavailable. The proportions held in the pH-affected carbonate-bound phase were generally only slightly higher than in the exchangeable phase. Environmental factors that tend to reduce soil pH will readily mobilize this fraction. Lead present in other chemical forms, such as Fe-Mn oxide bound (14%), organic matter bound (24%) and residual phase (17%) with very high stability and low solubility for biological activity are considered non-bioavailable.^18^ The proportions held in the organic matter-bound oxidizable phase were generally higher than in the reducible phase (Fe-Mn oxide bound) *(Table 6 and Figure 3)*. Changes in soil redox potentials and soil redox reactions will influence the release and retention of elements in these two phases. The 41% of lead in Dareta soil observed in the organic matter bound and residual fractions may likely be due to the fact that the organic matter and silicate in the residual fraction have high lead retention capabilities, since the tendency of such particulates to be adsorbed by the soil depends on the cation exchange capacity and organic composition of the soil.^21^ The residual phase fraction represent lead largely embedded in the crystal lattice of soil fraction and should not be available for remobilization except under very harsh conditions.^21^ The predominance of lead in organic matter bound fractions is in broad agreement with the results of Kabata-Pendias and Pendias for the affinity of Pb to soil organic matter.^32^ Ramos *et al*. found that most lead was associated with Fe-Mn oxides fraction in their study of Spanish soils.^35^ Onianwa reported higher levels in the Fe-Mn oxide bound fraction and residual fraction (37%) in Ibadan, Nigeria.^36^

### Potential bioavailability and mobility

With respect to bioavailability, various species of metals are more biologically available than others. The bioavailability and mobility of metals are closely related, the higher the concentration of mobile toxic metals in the soil column, the greater the potential for plant uptake, and animal/human consumption.^37^ Results of speciation analysis in this study shows that about 46% of lead in farm soils *(Table 7)* are associated with the non-residual phase, water soluble phase 10%, exchangeable phase 16%, and carbonate phase 19%. The MF values increased from 41.85 to 53.29 with an average of 45.51. Mobility factors between 10 and 48 have been described as considerably high.^23^, ^21^ In addition to reflecting the levels of water soluble, exchangeable and carbonate bound Pb, high MF values are an indication of liability and biological availability of the heavy metal, and shows the extent of the vulnerability of living organisms generally to the heavy metal.^21^ Although the soil Pb levels of farm soils in Dareta village are within the permissible limits, high bioavailability and mobility is a serious cause for concern as remobilization of the toxic metal into the other components (water, plant and biota) of the environment when physicochemical conditions are favorable cannot be ruled out. The calculated IACF for lead (*Table 6*) reflects the risk of contamination of farm soils by the heavy metal. The higher the levels of the mobilizable fractions (water soluble, exchangeable, bound to carbonates, Fe-Mn oxides bound and the organic matter bound fractions) in the soil, the higher the potential risk of contamination. The IACF values found were generally significant, with soil sample number 4 posing the highest risk. This result suggests that lead may easily be transferred into the food chain by plant uptake or leaching into ground water aquifers. At the moment, no standards for bioavailable lead have been developed, and its correlation with blood lead has not been demonstrated, nor that total lead is in fact a good predictor of blood lead levels, particularly in children.

### Interrelationships among soil pH, soil conductivity, extractable and total lead concentrations

The significant (p < 0.01) positive correlation observed between total lead concentration in soil and extractable lead concentration (bioavailable fraction, r = 0.977 and non-bioavailable fraction r = 0.988) and between total lead concentration in soil and soil conductivity (r = 0.966) indicates that an increase in total soil lead concentration is associated with an increase in extractable lead concentration and also that an increase in soil conductivity is associated with an increase in total soil lead concentration. Similar observations were made for contaminated and non-contaminated soils in Japan.^18^ The strong negative correlation between soil pH and extractable lead concentration (bioavailable fraction, r = −946 and non-bioavailable fractions r = −9.04) indicates that lead extractability decreased with increasing soil pH, as observed by many other investigators.^18^, ^38^ The positive correlations observed between soil conductivity and the extractable concentration (bioavailable fraction, r = 0.695 and non-bioavailable fraction r = 0.982) shows that increasing soil conductivity is associated with increasing extractability and by implication, bioavailability. The positive correlation between the concentration of bioavailable lead and the concentration of non-bioavailable lead in Dareta soil indicates that same source may be responsible for their presence at the determined concentrations.

## Conclusions

The audit of Pb in soil carried out in Dareta village three years after an elaborate remediation exercise following mass acute lead pollution and poisoning in the area shows a significant variation in soil Pb levels across the sampling stations, suggesting anthropogenic influence. Soil Pb levels in residential compounds and Dareta common areas exceeded both the Dutch target and intervention values and the USEPA limit for soil Pb levels in residential areas. The pollution intensity ranged from a strongly polluted to an extremely polluted soil environment, with an average accumulation factor of 2.83, indicating an approximately 183% increase in soil Pb levels compared to the remediation levels in 2010. Lead pollution of the Dareta ecological geochemical environment poses a range of moderate potential ecological risk—very high potential ecological risk to the other components of the environment. About 46% of the total Pb content of Dareta soil was found to be bioavailable, suggesting that transfer into the food chain through gradual leaching to ground water, uptake by plant or other solubilizing mechanism is eminent. Having acquired a remediation model and significant remediation capacity, the study recommends that Zamfara State authorities address the challenge of sustained periodic assessment and cleanup of the affected areas. Implementation of safer mining practices should be immediately enforced.

## References

[1] Tolulope AO (2004). Oil exploration and environmental degradation: the Nigeria experience. Environ Inform Arch.

[2] Ezike NN, Udiba UU, Ogabiela EE, Akpan NS, Odey MO, Inuwa B, Sule AM, Gauje B (2012). Assessment of the performance of primary effluent treatment plant of major tanneries in Kano, Nigeria. Trends Adv Sci Eng.

[3] Yahaya MI, Ezeh GC, Musa YF, Mohammad SY (2010). Analysis of heavy metals concentration in road sides soil in Yauri, Nigeria. Afr J Pure Appl Chem.

[4] Jung MC (2008). Heavy metal concentrations in soils and factors affecting metal uptake by plants in the vicinity of a Korean Cu-W mine. Sensors (Basel) [Internet].

[5] Uwah EI, Ndahi NP, Ogugbuaja VO (2009). Study of the levels of some agricultural pollutants in soils, and water leaf (Talinum triangulare) obtained in Maiduguri, Nigeria. J Appl Sci Environ Sanitat.

[6] Nriagu JO (1990). Global metal pollution poisoning the atmosphere. Environ.

[7] (2011). The world's worst toxic pollution problems: report 2011 [Internet].

[8] Israel DC, Asirot J (2002). Mercury pollution due to small-scale gold mining in the Philippines: an economic analysis.

[9] (2011). UNICEF programme cooperation agreement environmental remediation – lead poisoning in Zamfara. Final report September 2010–March 2011.

[10] Brown TJ, Hetherington LE, Hannis SD, Bide T, Benham AJ, Idoine NE, Lusty PA (2009). World mineral production 2003–07 [Internet].

[11] (2012). Lead poisoning from gold mining [Internet].

[12] Kabara AA (2014). Zamfara lead poison update: 735 child deaths recorded, 1450 on treatment-MSF. Leadership Online News Paper.

[13] Udiba UU, Ogabiela EE, Hammuel C, Magomya AM, Yebpella GG, Ade-Ajayi AF, Odey MO, Gauje B (2012). Post remediation assessment of contaminants levels in soil, Dareta Village, Zamfara, Nigeria. Trends Adv Sci Eng.

[14] (2010). Lead pollution and poisoning crisis environmental emergency response mission: Zamfara State, Nigeria September/October 2010 [Internet].

[15] (2003). Geographic data on Zamfara state.

[16] (2006). Social statistic in Nigeria.

[17] Begum A, Ramaiah M, Harikrishna, Khan I, Veena K (2009). Analysis of heavy metals concentration in soil and litchens from various localities of Hosur road, Bangalore, India. E-J Chem [Internet].

[18] Kashem MA, Singh BR, Kondo T, Imamul Huq SM, Kawai S (2007). Comparison of extractability of Cd, Cu, Pb and Zn with sequential extraction in contaminated and non-contaminated soils. Int J Environ Sci Technol [Internet].

[19] Tessier A, Campbell PG, Bisson M (1979). Sequential extraction procedure for the speciation of particulate trace metals. Anal Chem [Internet].

[20] Hakanson L (1980). An ecological risk index for aquatic pollution control: a sedimentological approach. Journal of Water Research.

[21] Ogunfowokan AO, Oyekunle JA, Durosinmi LM, Akinjokun AI, Gabriel OD (2009). Speciation study of lead and manganese in roadside dusts from major roads in Ile-Ife, south western Nigeria. Chem Ecol [Internet].

[22] Uzairu A, Harrison GF, Balarabe ML, Nnaji JC (2009). Concentration levels of trace metals in fish and sediment from Kubanni River, northern Nigeria. Bull Chem Soc Ethiop [Internet].

[23] Yusuf KA (2007). Sequential extraction of lead, copper, cadmium and zinc in soils near Ojota Waste Site. J Agron.

[24] Qingjie G, Jun D, Yunchuan X, Qingfei W, Liqiang Y (2008). Calculating pollution indices by heavy metals in ecological geochemistry assessment and a case study in parks of Beijing. J China Univ Geosci [Internet].

[25] Chatterjee M, Silva Filho EV, Sarkar SK, Sella SM, Bhattacharya A, Satpathy KK, Prasad MV, Chakraborty S, Bhattacharya BD (2007). Distribution and possible source of trace elements in the sediment cores of a tropical macrotidal estuary and their ecotoxicological significance. Environ Int [Internet].

[26] Malik RN, Husain SZ, Nazir I (2010). Heavy metal contamination and accumulation in soil and wild plant species from industrial area of Islamabad, Pakistan. Pak J Bot.

[27] Aydinalp C (2009). Concentration and speciation of Cu, Ni, Pb and Zn in cultivated and uncultivated soils. Bulg J Agric Sci.

[28] Ghosh M, Singh SP (2005). A review on phytoremediation of heavy metals and utilization of its byproducts. Appl Ecol Environ Res.

[29] Mico C, Peris M, Sanchez J, Recatala L (2006). Heavy metal content of agricultural soils in a Mediterranean semiarid area: the Segura River Valley (Alicante, Spain). Span J Agric Res.

[30] (2000). Dutch target and intervention values, 2000 (the New Dutch List) [Internet].

[31] Ogbonna PC, Okeke VI (2011). Metal content of soil and macronutrient of Gmelina leaves in Umuahia, Nigeria. J Appl Sci Environ Sanit.

[32] Yoon J, Cao X, Zhou Q, Ma LQ (2006). Accumulation of Pb, Cu, and Zn in native plants growing on a contaminated Florida site. Sci Total Environ [Internet].

[33] Kabata-Pendias A, Pendias H (1992). Trace elements in soils and plants.

[34] Moore F, Forghani G, Qishlaqi A (2009). Assessment of heavy metal contamination in water and surface sediments of the Maharlu Saline Lake, SW Iran. Iran J Sci Technol Trans A.

[35] Ramos L, Hernandez LM, Gonzales MJ (1994). Sequential fractionation of copper, lead, cadmium and zinc in soils from or near Doñana National Park. J Environ Qual.

[36] Onianwa PC (2001). Roadside topsoil concentrations of lead and other heavy metals in Ibadan, Nigeria. Soil Sediment Contam [Internet].

[37] Zimmerman AJ, Weindorf DC (2010). Heavy metal and trace metal analysis in soil by sequential extraction: a review of procedures. Int J Anal Chem [Internet].

[38] Singh BR, Narwal RP, Jeng AS, Almas A (1995). Crop uptake and extractability of cadmium in soils naturally high in metals at different pH levels. Commun Soil Sci Plant Anal [Internet].

